# Note on Some of the Medicinal Uses, as an Internal Remedy Principally, of the Muriate of Ammonia

**Published:** 1855-08

**Authors:** H. A. Ebden


Note on some of the Medicinal Uses, as an Internal Remedy principally, of the Muriate of Ammonia. By H. A. Ebden, M. D.

The author has written this paper under the conviction that the hydroclorate of ammonia (sold in the native Indian bazaars under the name of "nausaada") is not sufficiently appreciated by English practitioners as an internal remedy, and states his conviction, after a full trial of its merits, that it is a very valuable and powerful remedy for the relief of neuralgic pain generally. The diseases in which he states that he has given it with success are facial neuralgia, tic-douloureux, nervous headache, toothache, clavus hystericus, sciatica, and neuralgic dysmenorrhœa. He usually prescribes it in doses of twenty-five to thirty-five grains in an ounce of mint water or camphor mixture every twenty minutes for three doses. The second dose is usually sufficient for the relief of the immediate pain; but he has observed that where it has been necessary to repeat and continue the doses, the patient has, in many instances, afterwards enjoyed a comparative immunity from the recurrence of pain; and he has, therefore, been led to continue the exhibition of the salt systematically, at six or eight hours' interval, for seven days. Externally applied, too, in strong solution and hot, under oiled silk, he states he has found it an excellent palliative to neuralgic pain. The other uses of the medicine he thus enumerates. In doses of five, ten, and fifteen grains, twice and thrice a day, in bitter infusions, it proves alterative, and stimulates the action of the liver in cases where the exhibition of mercurials is undesirable. Externally, in combination with nitre and sulphur in equal proportions, the powder being moistened with vinegar, he recommends it in ringworm and impetiginous skin affections; and it is thus, he believes, used by the natives of Lower Bengal. In very strong solutions, applied hot, he recommends its use as a topical anodyne in various painful affections.—*Medico-Chir. Review.*

